# Strengthening or Restricting? Explaining the Covid-19 Pandemic’s Configurational Effects on Companies’ Sustainability Strategies and Practices

**DOI:** 10.1177/00076503221134100

**Published:** 2022-11-29

**Authors:** Ralph Hamann, Alecia Sewlal, Neeveditah Pariag-Maraye, Judy Muthuri, Kenneth Amaeshi, Ijeoma Nwagwu, Jenny Soderbergh

**Affiliations:** 1 University of Cape Town, South Africa; 2 University of Mauritius, Reduit, Mauritius; 3 Nottingham University Business School, UK; 4 University of Edinburgh Business School, UK; 5 Pan-Atlantic University, Lagos, Nigeria

**Keywords:** Africa, Covid-19, crisis, strategy, sustainability, threat-rigidity

## Abstract

We explore the Covid-19 pandemic’s impact on companies’ sustainability strategies and practices. Prior research has identified a number of factors that shape such effects, including crisis severity, resource slack, and prior investments, but their interactions have not been given much attention. We thus collected qualitative data on 25 companies in four African countries, which we analyzed inductively and iteratively through cross-case comparison and with fuzzy set Qualitative Comparative Analysis. We identify two pathways associated with strengthening responses (“building on strengths” and “governance gap-filling”) and three associated with restricting responses (“hard hit,” “low-road business-as-usual,” and “bunkering down”). Our findings enhance our understanding of organizational responses to crises by attending to configurational effects, by elaborating the role of prior sustainability investments, and by foregrounding the relevance of governance contexts. We describe implications for future research and managers, investors, and sustainability initiatives such as the United Nations Global Compact.

Covid-19 has been a “societally disruptive extreme event. . . with profound implications for the role of business in society” (Brammer et al., 2020, p. 494). One of these implications is the effect of the pandemic on companies’ commitments to addressing longer term systemic challenges, such as poverty, inequality, and climate change, through their sustainability strategies. Companies may see the crisis as a reason to cut costs and restrict their sustainability strategies, which may enhance their own short-term financial prospects but reduce the well-being and social–ecological resilience of the communities and societies they operate within, “undermining planetary survival” (Adams & Abhayawansa, 2021). Alternatively, companies may see in the crisis motivation and opportunity to enhance their sustainability commitments, based on the pandemic foregrounding the “interdependencies among the organizational system and the broader social and environmental systems in which the organization is embedded” (DesJardine et al., 2019, p. 1438).

We build upon a long-standing scholarly conversation on whether crises catalyze organizational innovation or rigidity (e.g., Chattopadhyay et al., 2001; Dutton, 1986; James et al., 2011; Ocasio, 1995; Osiyevskyy & Dewald, 2018; Sarkar & Osiyevskyy, 2018; Shimizu, 2007; Staw et al., 1981) as well as a more nascent focus on crises’ effects on companies’ sustainability strategies and practices (Bansal et al., 2015; M. L.Barnett et al., 2015; Delmas & Pekovic, 2015; Panwar et al., 2015). This prior work has identified a number of factors that may shape such effects, including organizations’ resource slack, institutional context, and prior sustainability-related investments. It has also suggested that there are “aggregate effect[s]” (Panwar et al., 2015, p. 216) between these various factors, but such interactions and the resulting configurations of contingent factors have as yet been given limited attention in both the broader literature on crisis and organizational change and the more specific conversation on sustainability responses to crises. Such interaction effects are important to explore in the context of the Covid-19 pandemic, which has been a particularly severe and extensive crisis with significant and diverse effects in different places, sectors, and companies (e.g., Adams & Abhayawansa, 2021; Bacq & Lumpkin, 2020; Bapuji, Patel, et al., 2020; Brammer et al., 2020; Crane & Matten, 2020; Howard-Grenville, 2020, 2021; Rouleau et al., 2020; Seidl & Whittington, 2020). Hence, we ask, how has the Covid-19 pandemic affected companies’ sustainability strategies and practices?

We explore this question by means of qualitative data collected on 25 companies in four African countries—Kenya, Mauritius, Nigeria, and South Africa—which we analyze inductively and iteratively by complementing cross-case comparison (Eisenhardt, 1989, 2021) with fuzzy set Qualitative Comparative Analysis (fsQCA; Misangyi et al., 2017; Ragin, 1999, 2012; Schneider & Wagemann, 2010; Slager et al., 2021). We discover how different configurations of crisis severity, resource slack, sustainability maturity, and two aspects of institutional context contribute to motivating and enabling either restricting or strengthening outcomes. Specifically, we identify two dynamics associated with strengthening responses (“building on strengths” and “governance gap-filling”) and three associated with restricting responses (“hard hit,” “low-road business-as-usual,” and “bunkering down”).

Our analysis demonstrates the benefits of a configurational analytical approach to developing a more diverse and nuanced picture of crisis impacts on organizations; it elaborates how prior investments shape crisis responses; and it highlights the role of governance contexts. Our findings invite further research with smaller and larger samples, and they have practical implications for managers, investors, and sustainability initiatives such as the United Nations Global Compact.

## Theoretical Background

### Crises as Catalysts of Change Versus Rigidity

There is long-standing scholarly interest in whether crises and threats constrain or enable organizational change and innovation (e.g., James et al., 2011; Ocasio, 1995; Osiyevskyy & Dewald, 2018; Sarkar & Osiyevskyy, 2018). Influential early contributions have highlighted the complexity of organizations’ responses to crises, requiring consideration of multiple levels of analysis and their interactions, as well as a corresponding combination of diverse theories. For example, Staw et al. (1981) develop a multilevel argument integrating theories on individual psychology and group and organizational behavior, and Ocasio (1995) adds prospect theory and neo-institutional theory, among others.

On the one hand, adversity associated with crises is seen as a catalyst for organizational change and learning. According to the behavioral theory of the firm (Cyert & March, 1963), crises and the associated failures to fulfill aspirations motivate problem-solving, risk-taking, and organizational learning, as long as the stress is not too extreme. Crises catalyze managerial attention and focus (Dutton, 1986) as well as positive leadership if the leader frames the crisis as an opportunity rather than a threat (C. K.Barnett & Pratt, 2000; Chattopadhyay et al., 2001; James et al., 2011). Positive organizational changes in response to crises benefit from resource slack, in line also with the resource-based view of the firm (Pitelis, 2007; Williams et al., 2017).

In contrast, Staw et al. (1981) develop the threat-rigidity thesis, in which adversity leads to restricted information processing, constriction of control, and an emphasis on efficiency so as to conserve resources. Unless the crisis involves only incremental and well-known changes, these threat-rigidity responses are often maladaptive because they prevent the kind of innovation, flexibility, and learning required to respond to a radically changing environment. Empirical support for the threat-rigidity thesis includes studies showing that leaders engaged in more directive behaviors in response to the 2008 financial crisis (Stoker et al., 2019) and the Covid-19 lockdowns (Garretsen et al., 2022); that increasing environmental hostility diminishes firms’ entrepreneurial orientation (Kreiser et al., 2020); and that threat-rigidity leads to collective moral disengagement (Welbourne Eleazar, 2022).

Ocasio (1995) seeks to reconcile these opposing change and rigidity perspectives by bringing to bear and connecting cognitive and institutional theories. He argues that adversity increases managers’ risk-taking behaviors (following prospect theory; Kahneman & Tversky, 1979) while simultaneously narrowing their attention on the specific problems related to the crisis and a focus on “solutions that are consistent with the core cultural assumptions” within and beyond the organization (Ocasio, 1995, p. 314). Chattopadhyay and colleagues (2001) find empirical support for this combination of prospect theory and the threat-rigidity thesis.

Others have sought to reconcile or connect theories of crisis-induced change and rigidity by focusing on contingent or moderating factors (Osiyevskyy & Dewald, 2018; Sarkar & Osiyevskyy, 2018; Shimizu, 2007). Some of these factors are associated with crisis characteristics. As noted earlier, if crisis severity and uncertainty are high, organizations will likely be more conservative and constricted in their attention, whereas if the severity is more moderate, they can engage in exploration and learning (Garretsen et al., 2022; March & Shapira, 1987; Shimizu, 2007). Similarly, a crisis brought about by a sudden, “deviant” event is more likely to catalyze rigidity responses, whereas a slower-onset crisis enables managers to engage in problem-solving and innovation behaviors with others in the firm (Osiyevskyy & Dewald, 2018). Other contingent factors relate to organizational features. Over and above resource slack, if a company has some existing “familiarity of innovation” (Sarkar & Osiyevskyy, 2018), this will likely enable such innovation during a crisis (building on Ocasio, 1995). Finally, contingent factors may relate to individual characteristics. The important potential role of leaders’ cognitive frames (James et al., 2011) has already been mentioned, and in a similar vein, employees’ creativity is more likely to increase in a crisis if they have a “growth mindset” (Jeong et al., 2022).

All in all, however, these diverse contingencies are still up for debate, both theoretically and empirically (Connelly & Shi, 2022; Kreiser et al., 2020; Osiyevskyy & Dewald, 2018). We suggest that in part this is because these contingent factors have largely been considered in isolation of each other, whereas there are likely configurations of contingent factors that interact to give rise to diverse organizational responses to crises.

### Change Versus Rigidity With Respect to Sustainability Strategies

The research outlined earlier has largely focused on organizational responses in broad terms, such as strategic survival and innovation. It is thus relevant to consider the diverse ways, in which our more specific focus on sustainability strategies and practices relates to such broader strategic objectives. There are, broadly speaking, two dimensions to this relationship, both objectively speaking and in managers’ cognitive frames (Hahn et al., 2014). One of them is synergistic, in that sustainability strategy may be seen to directly contribute to the broader objectives of survival, growth, and innovation as part of a “business case” framing. The other highlights the existence of tensions given that there are possible trade-offs between a firm’s economic objectives and social and environmental ones, including temporal trade-offs between short-term survival and longer term sustainability benefits (Bansal & DesJardine, 2014). Furthermore, the relationship between sustainability and a firm’s broader strategy is likely shaped by firm-internal organizational dynamics, such as sustainability managers’ experience and influence in the organization (Ocasio, 1995).

Building on the literature reviewed earlier, we may expect that a firm’s sustainability strategy and practices will be constricted or emboldened as part of the broader organizational response, depending on contingent factors such as crisis severity and speed. Some aspects of a sustainability response, however, will be more specific to this strategic domain and how it manifests within the firm. If sustainability is seen to be vital to the company’s “core” strategy and if the existing sustainability group has influence in the organization, sustainability strategy and practices are less likely to be constricted and may even expand during a crisis. On the contrary, if sustainability is seen as separate from core strategy within a firm, with only indirect or longer term relevance, it is more likely to succumb to rigidity and efficiency-focused responses by the organization.

These broad theoretical expectations are largely borne out by an emerging scholarly conversation that focuses on crises’ impacts on firms’ sustainability strategies and practices, catalyzed especially by the financial crisis of 2008 (Bansal et al., 2015; M. L.Barnett et al., 2015; Panwar et al., 2015). A common point of departure in this work is that a crisis will curtail resources, which in turn leads to a restriction of firms’ sustainability efforts. This effect is mitigated by available resource slack, and it also varies across different sustainability efforts. Panwar and colleagues (2015) find that firms responded to the financial crisis of 2008 by restricting community initiatives, which are described as peripheral strategies, more than core strategies related to environmental initiatives. Such core initiatives “would have absorbed a significant amount of firm’s resources [pre-crisis], making it difficult to downscale” (Panwar et al., 2015). Furthermore, this negative effect on peripheral initiatives is especially pronounced in dynamic and unpredictable contexts. This leads the authors to see “firms’ sustainability behaviour as an aggregate effect of resources. . . and competitive context” (Panwar et al., 2015, p. 216).

Bansal and colleagues (2015) arrive at similar findings in a sample of corporations responding to the 2008 financial crisis. Most corporations reduced their commitments to corporate social responsibility (CSR) during the crisis, but this was more pronounced for tactical CSR (including in particular community contributions) than for strategic CSR, which includes environmental and human rights policies and practices. Such strategic CSR “requires long time horizons, large resource commitments and significant structural reform [. . .and] is thus more difficult to implement and reverse” (Bansal et al., 2015, p. 76). Furthermore, those firms with higher financial performance prior to the crisis have slack resources that not only contribute to their financial resilience but also help them maintain their strategic CSR commitments. Such firms, the authors suggest, also have a helpful “long-term focus” (Bansal et al., 2015, p. 77).

Bansal and colleagues refer to some cases, where companies with resource slack even enhanced CSR commitments during the crisis, but this is not given much explicit attention. Delmas and Pekovic (2015) also find that some firms “swim against the stream” and invest resources in innovation during a crisis, with a specific focus on resource efficiency measures. They argue that this is more likely for larger and more vertically integrated firms that have developed relevant knowledge and capabilities by having adopted environmental standards and invested in R&D. This is in line with prior work on “the familiarity of innovation” as a contingency factor in organizational threat-rigidity responses (Ocasio, 1995; Sarkar & Osiyevskyy, 2018). While these are pertinent enabling factors, it is not clear why such firms might use a crisis to actually increase sustainability investments.

### The Covid-19 Pandemic

The Covid-19 pandemic has been a kind of “meta-crisis” in that it combined health, economic, and social dimensions, as well as adversity as both event and process (Sarkar & Osiyevskyy, 2018; Shepherd & Williams, 2020). It has severely affected both supply (Sarkis, 2021; Yu et al., 2022) and demand (Wang et al., 2020). Yet these restrictions had varying salience and severity in different sectors, with sectors such as hospitality and air travel particularly affected. They also varied across space and time, with government “lockdown” regulations imposed at varying levels of severity by different governments and also at different times. The pandemic has had a significant influence on longer term social and organizational dynamics and thus becomes a theoretically relevant object of analysis in its own right, and this is becoming evident in a flurry of scholarly work also in organization and management studies (e.g., Adams & Abhayawansa, 2021; Bacq & Lumpkin, 2020; Bapuji, Patel, et al., 2020; Brammer et al., 2020; Crane & Matten, 2020; Howard-Grenville, 2020, 2021; Rouleau et al., 2020; Seidl & Whittington, 2020).

The nascent management literature on Covid-19 suggests significant uncertainty and even contradictory expectations with regard to its effect on companies’ sustainability strategies. On the one hand, the pandemic has created severe resource constraints, especially in sectors directly hit by government lockdown regulations, such as travel and tourism. The need to stay afloat in very difficult operating and market conditions has brought about concerns that companies “deprioritise costly environmentally sustainable policies and initiatives, undermining planetary survival” (Adams & Abhayawansa, 2021; see also Amankwah-Amoah, 2020). Curtailed sustainability efforts are thus part of a broader set of “retrenchment” responses (Wenzel et al., 2021). Such resource-related concerns are compounded by possible effects of the Covid-19 pandemic on the attentional time horizons of managers: “the high uncertainty combined with the severity of impacts of the pandemic has created an impetus to take quick short-term action that often conflict with the need to consider the long-term viability of our economy and society” (Slawinski in Carmine et al., 2021, p. 139).

On the contrary, the crisis may have fostered managerial attention to the interdependence of business and society, and to longer term systemic crises such as climate change and inequality (Adams & Abhayawansa, 2021; Bacq & Lumpkin, 2020; Bapuji, de Bakker, et al., 2020; Brammer et al., 2020; Hahn in Sharma et al., 2021). Brammer and colleagues (2020, p. 501) argue the “crisis has demonstrated how collaboration among government, business, and civil society could work and an unexpected appetite for responding to the crisis in a relatively selfless and community-spirited way”. Theoretically speaking, the pandemic may have created the institutional turbulence for the reappraisal of established ideas and the generation and adoption of new ideas (Blyth & Mark, 2002; Fligstein, 1991) related to the business and society interrelationship.

In summary, we argue that it is important to understand the effects of crises on companies’ sustainability strategies and this is especially so for the Covid-19 pandemic as a particularly formative crisis with longer-term repercussions. This ambition should build upon a long-standing scholarly conversation on the role of crises in organizational change, but also consider the domain-specific characteristics of firms’ sustainability strategies and practices. Prior work has identified a number of factors that may shape such crisis effects, including resource slack, institutional context, and prior investments. It has also suggested that there are “aggregate effect[s]” (Panwar et al., 2015, p. 216) between these various factors, but such interactions have as yet been given limited attention in both the broader literature on crisis and organizational change as well as the more specific conversation on sustainability responses to crises.

## Method

We collected qualitative data on 25 companies in four African countries: Kenya, Mauritius, Nigeria, and South Africa. We focused on an African context in response to Howard-Grenville and colleagues’ (2019, p. 363) suggestion to attend especially to “those most affected by the [sustainable development] problems.” We also seek to address the relative paucity of management research on business sustainability strategies or on the Covid-19 pandemic in African settings, despite invitations to give more emphasis to such contexts (George et al., 2016; Khayesi & George, 2011), especially in studies on business and society (Adeleye et al., 2020; Kolk & Rivera-Santos, 2018). We included four countries that represent different regions of the continent and also different levels of socioeconomic and institutional development (Mo Ibrahim Foundation, 2020). This is pertinent also because of the possible role of companies’ institutional context in shaping organizational responses to crises (Ocasio, 1995) and specifically sustainability-related responses (Panwar et al., 2015). Finally, our country focus was influenced by the fact that each of our team members was based or had significant knowledge and relationships in one of these countries.

In each country, we selected between four and 10 companies, seeking to include companies from primary, secondary, and tertiary sectors, and including a range of sizes, from domestic subsidiaries of large multinational corporations to family-owned medium-sized enterprises. The different sectors ensure that we have different degrees of direct impact from the crisis, including for instance hotel and travel companies (high direct impact), on the one hand, and retail and finance companies (relatively less direct impact), on the other. We also have a spread of resource slack and prior sustainability commitment within our sample, and as mentioned, the four countries represent different degrees of institutional stability. We thus have salient diversity in our sample (Eisenhardt, 1989; Ragin, 1999) on factors that likely affect organizational responses to crises.

### Data Collection and Preliminary Analysis

We collected data on the case study companies, as well as their four country settings, by means of interviews, online group discussions, and document analysis, in line with recommendations for case study research (Eisenhardt, 1989; Yin, 1994). Our main source of data was two rounds of interviews with the most senior sustainability manager in each company. We selected these interviewees because they had the most immediate knowledge of how their companies’ sustainability strategies and practices were affected by the pandemic.

After some preliminary conversations with some of our participants in May-June 2020, which helped us conceptualize and plan our research, we conducted our first round of interviews between August and December 2020. The interviews were between 45 and 90 min long and were semi-structured, making use of an interview protocol with two sections. The first section focused on the process through which the company had established its sustainability strategy, including the identification of measurable targets. It also included questions on how this strategy was affected by the Covid-19 pandemic. The second section focused on opportunities and challenges encountered in strategy implementation and goal attainment, and again this included questions on the impact of Covid-19. Our interviews were all conducted using online platforms (commonly Zoom or MS Teams). Such online platforms became a more efficient and effective form of data collection than initially expected. (For dedicated discussions of the use of online platforms for data collection, see Archibald et al., 2019; Gray et al., 2020). We augmented these first round interview data with information from company reports and websites, with a focus on annual reports, sustainability reports, and / or integrated reports. We also conducted 13 interviews with stakeholders in government, business associations, nongovernmental organizations (NGOs), or consulting organizations, who could provide us with information and insight on our country contexts as well as some cross-cutting perspectives about our research question across companies and sectors.

We conducted a first round of analysis on these data, with an emphasis on participants’ own meanings (Glaser & Strauss, 1967). This first stage of analysis included the writing of preliminary case reports (Eisenhardt, 1989; Yin, 1994) on each company. Cross-case comparison of these company cases gave rise to country-level analysis reports that highlighted similarities and differences between the companies in each country. We then engaged in numerous discussions across the research team, facilitated by the use of various tables (Miles et al., 2014), to identify key themes and patterns emerging across our four countries. This resulted in a preliminary analysis report, which we used as a platform for our second round of interviews.

Our preliminary analysis gave rise to an initial categorization of companies’ pre-crisis sustainability commitments, based especially on the degree to which they had moved from merely reporting on sustainability themes to the transparent use of measurable and ambitious targets. Our definition and analysis of firms’ sustainability strategies and practices thus foreground the “strategic” (Bansal et al., 2015) or “core” (Panwar et al., 2015) dimensions of companies’ sustainability commitments, although this did not prevent us from also considering more “peripheral” (Panwar et al., 2015) or philanthropic aspects. Furthermore, our emphasis on companies articulating targets and measuring performance against contextually relevant social and ecological sustainability issues (Bertels & Dobson, 2017) was motivated among other things by the need to consider both the means and the ends of sustainability performance (Halme et al., 2020). Our preliminary analysis also identified three ways in which companies’ sustainability efforts were affected by the pandemic: *restricting*, or scaling down due to budget cuts or similar constraints created by the crisis; *re-focusing* on new priorities, such as, for example, humanitarian needs such as hunger relief or on employee wellness; and *reemphasizing*, based on managers seeing in the pandemic a motivation for greater sustainability commitment.

The second round of interviews was conducted in March and April 2021. These interviews focused on two dimensions. First, we asked interviewees for an update on how the ongoing Covid-19 crisis had impacted their company and specifically their sustainability efforts. This was motivated by our intention to capture not just the immediate reactions to the pandemic (i.e., in the first half of 2020) but also those changes that were visible a year or so into the crisis. Second, we probed more deeply into some of the key themes that emerged in the analysis of our first round data, including the degree or manner, in which the company was setting ambitious targets and measuring performance. We mentioned the three kinds of sustainability strategy impacts noted earlier and explored whether or how these were manifest in the company. (This latter component was included at the end of the interview, so as to mitigate bias.) These interviews thus led to a number of adjustments to our preliminary analysis, such as the inclusion of a fourth category of Covid-19 response (initially called “experimenting” and later, “innovating”).

Finally, we organized two online discussions to share our emerging findings and arguments, and to solicit feedback. For the first, we invited only our interviewees to enable a more in-depth discussion. This meeting had 20 participants. The second online discussion was co-hosted with the United Nations Global Compact (UNGC) Local Networks in each of our four countries, and this invitation was more broadly distributed. This meeting had about 40 participants, including the executive directors of the Local Networks in our four countries. We also benefited from numerous preparatory conversations with colleagues from the UNGC. The primary benefit from these online discussions and the associated conversations was to clarify and validate our emerging codes and themes and their interrelationship. Table 1 provides an overview of our case study companies and our corresponding data.

**Table 1.** Overview of Our Cases and Corresponding Data.

Country	Company	Sector	Data
South Africa	ZA1	Food and beverage	2 interviews; online discussions; corporate reports
	ZA2	Energy and chemical	2 interviews; email correspondence; corporate report
	ZA3	Retail	2 interviews; corporate reports
	ZA4	Hospitality and tourism	2 interviews; online discussion; corporate reports
	ZA5	Insurance	2 interviews; corporate reports
	ZA6	Transportation and logistics	2 interviews; email correspondence; corporate reports
			*Country context*: Interviews and email correspondence with country-level stakeholders: business association (2 interviews, plus online discussion); UNGC Local Network; various reports from government agencies, NGOs, and media
Mauritius	MU1	Hospitality and tourism	2 interviews; online discussion; corporate reports
	MU2	Manufacturing / consumer goods	2 interviews; corporate reports
	MU3	Agriculture / food processing	2 interviews; online discussions; corporate reports
	MU4	Financial services	2 interviews; corporate reports
	MU5	Manufacturing	2 interviews; online discussions; corporate reports
	MU6	Real estate / investment	2 interviews; corporate reports
	MU7	Hospitality and tourism	2 interviews; corporate reports
	MU8	Manufacturing / consumer goods	2 interviews; corporate reports
	MU9	Financial services	2 interviews; corporate reports
	MU10	Food and beverage	2 interviews; online discussions; corporate reports
			*Country context*: Interviews and email correspondence with country-level stakeholders: business association; government agency; UNGC Local Network; various reports from government agencies, NGOs, and media
Nigeria	NG1	Manufacturing / consumer goods	2 interviews; UNGC dialogue; corporate reports
	NG2	Mining and manufacturing	2 interviews; corporate reports
	NG3	Telecommunications	2 interviews; corporate reports
	NG4	Food and beverage	1 interview; corporate reports
			*Country context*: Interviews and email correspondence with country-level stakeholders: business association; government agency; UNGC Local Network; various reports from government agencies, NGOs, and media
Kenya	KE1	Financial services	2 interviews; corporate reports / websites
	KE2	Financial services	2 interviews; online discussion; corporate reports / websites
	KE3	Financial services	1 interview; corporate reports
	KE4	Manufacturing / consumer goods	2 interviews; online discussion; UNGC dialogue; corporate reports
	KE5	Aviation	2 interviews; corporate reports
			*Country context*: Interviews and email correspondence with country-level stakeholders: business association (3 interviews); civil society; UNGC Local Network; various reports from parliament, national and county government, government agencies, NGOs, and media
Total			52 company interviews (recorded and transcribed); total duration: about 3400 minutes 13 national stakeholder interviews (recorded and transcribed); total duration: about 700 minutes Online discussion with interviewees (90 minutes) and online UNGC dialogue (90 minutes) (recorded) Various corporate reports and other documents, including media

*Note.* UNGC = United Nations Global Compact; NGO = Nongovernmental Organization.

### Qualitative Comparative Analysis

Although our cross-case analysis helped us identify outcomes and explanatory factors, which we could begin to relate back to the literature, we struggled to identify clear patterns in our 25 cases. We suspected that this was due to different interactions between the various factors going well beyond the “aggregate effect of resources. . . and competitive context” (Panwar et al., 2015). In other words, we needed a more rigorous consideration of how configurations of factors shape crisis-induced effects in companies’ sustainability strategies, with different configurations possibly giving rise to similar outcomes. Our response was to recode the data and then analyze them using fsQCA, which, among other things, is particularly appropriate when seeking to understand the (possibly equifinal) effects of configurations of factors (Fiss, 2011; Ragin, 1999). It uses Boolean algebra to identify how different configurations of cases’ attributes are associated with specific outcomes. We use the fsQCA in a complementary manner rather than a stand-alone analytical technique. Not only do we use our cross-case analysis described earlier to determine the attributes and their measurement as inputs for the fsQCA, but we also use the configurations identified in the fsQCA to return to our qualitative data to elaborate the dynamics giving rise to different outcomes. Our approach was hence to use the “QCA’s configurational analysis. . . in an iterative dialogue with cases” (Misangyi et al., 2017, p. 271).

Our fsQCA analysis initially considered four attributes, which we identified in our literature review and in our preliminary data analysis. *Crisis severity* addresses the degree to which a company was directly affected by the Covid-19 pandemic and this had a strong sectoral dimension, as tourism and travel companies were more directly impacted by government lockdowns and travel restrictions than, say, finance companies. *Resource slack* addresses the degree to which a company had savings or access to credit to “weather the storm.” *Sustainability maturity* refers to the degree, to which a company had advanced its sustainability strategy prior to the crisis, and we assessed this with regard to, for example, a company’s commitment to transparent and ambitious sustainability targets. To categorize each of our cases with regard to these three attributes, we relied on our interview and archival data. We used a 4-point scale so as to avoid calibrated values of 0.5 and the associated ambiguity in the fsQCA.

Our fourth attribute, *governance stability and effectiveness* (henceforth *governance stability*), assesses the stability and predictability of a company’s institutional context, and whether this context ensures the provision of public goods and services. To measure this attribute, we relied on the Mo Ibrahim Index, a measure of countries’ governance stability and effectiveness (Dassah, 2015; Farrington, 2009; Mo Ibrahim Foundation, 2020). When we conducted the fsQCA analysis using these four attributes, we identified a “deviant” configuration in our results, which suggested the existence of salient dynamics related to the institutional context that were not included among our list of attributes. Specifically, the configuration suggested that there were pertinent institutional differences between Kenya and Nigeria that were not encapsulated by the *governance stability* attribute. By analyzing the differences between these countries, we came to the conclusion that not only governance stability but also changes over time in governance stability and effectiveness may be playing a role. (Kenya experienced an improvement in governance stability, in contrast to Nigeria, Mauritius, and South Africa.) We thus included a fifth attribute, *governance trend*, to capture this aspect and to address the contradictions identified in the preliminary fsQCA (for the technical underpinnings of this process, see Wagemann & Schneider, 2012). To codify this attribute, we used an additional measure provided by the Mo Ibrahim Index, which assesses countries based on changes in their governance stability and effectiveness over the preceding 10- and 5-year periods (as summarized in Table 2) (Mo Ibrahim Foundation, 2020). Including this fifth attribute also improved our fsQCA’s inclusion, consistency, and coverage parameters for our solutions.

**Table 2.** Coding for the Attributes and Outcome in Our Fuzzy Set Qualitative Comparative Analysis.

Attribute	Measure	Source	Value definitions
Crisis severity	The impact of COVID based on both internal and external factors at play	Our coding of companies against this condition was based on data from our interviews and company reports, as well as country-level analyses of different sectors, because the pandemic’s economic impacts on a company was significantly shaped by the sector. For example, some of the hotel or travel companies in our sample closed all of their operations and lost all revenue for the entire research period, whereas some companies in other sectors, such as retail or technology, hardly had any interruptions to their business or even experienced	Coded as follows: 1. no negative impact, or some positive impact 2. limited impact (e.g., short-term closure of operations) 3. significant impact (e.g., partial or medium-term closures of operations) 4. severe impact (e.g., extended closure of operations).
Resource slack	The company’s resource slack, including savings and access to credit	This coding needed to respond to significant company-level idiosyncrasies, given that some companies had more cash reserves or better credit scores than others. We also considered some country-level patterns, because companies in Mauritius generally had better access to government grants and loans in response to the pandemic than those in Kenya and Nigeria.	Coded as follows: 1. no or very limited resources 2. limited access to internal or external resources 3. some access to either internal or external resources 4. significant access to both internal and external resources
Sustainability maturity	This sought to capture the degree and depth to which sustainability had been embedded as a strategic objective in the firm	We initially explored the possibility of using related stage-models in the literature (e.g., Dyllick & Muff, 2016) for this purpose. But eventually—based on our preliminary data analysis and validation process—we developed our own categorization and corresponding coding process, as outlined on the right.	Coding as follows: 1. “learners” are companies that have only quite recently embarked on their “sustainability journey”, as was evident for example in their recent establishment of a sustainability strategy and department;
			2. “reporters” are companies that have made progress in reporting internally and externally on sustainability themes, but this does not include explicit, measurable, and ambitious targets; 3. “target-setters” publish comprehensive reports but include explicit targets that are also linked to managers’ key performance indicators (KPIs) and compensation; and 4. “leaders” set targets linked to KPIs, but they distinguish themselves by playing a leadership role in collaborating with government and other stakeholders to advance sustainability at the sector or system level
Governance stability	Governance stability and effectiveness, as measured by the Mo Ibrahim Index	The Mo Ibrahim Index assesses the governance stability and effectiveness of a country based on the provision of political, social and economic goods to which its citizens are entitled. Countries are rated based on several factors resulting in an overall score that ranges from 0 to 100.	The country scores between 0 and 100 were calibrated using the Direct Method (Duşa, 2019) with exclusion set at 25, cut-off at 50, and inclusion at 75.
Governance trend	Governance stability trend over a 10 year period, as categorized by the Mo Ibrahim Index Trend	The Mo Ibrahim Index Trend measure considers changes in each country’s governance stability and effectiveness in the preceding 10 years. The change in the Mo Ibrahim Index score over the 10-year period is categorized according to trends ranging from “Increasing Deterioration” to “Increasing Improvement”.	Each country was scored on a scale of 1 to 6 based on the governance trends identified in the Mo Ibrahim Index report. The countries were coded as follows: 1. “Increasing Deterioration” over the last ten years, with the rate of decline increasing over the last 5 years 2. “Slowing Deterioration” over the last ten years, but the rate of decline is slowing over the last 5 years 3. “Warning Signs,” i.e., some positive change or no change over the last ten years, but recent decline over the last 5 years 4. “Bouncing Back,” i.e., some negative change or no change over last ten years, but recent improvement over the last 5 years 5. “Slowing Improvement” over the last ten years, with the rate of improvement slowing over the last 5 years 6. “Increasing Improvement” over the last ten years, with the rate of improvement increasing over the last 5 years
Sustainability strategic response	This refers to how a company’s sustainability efforts and strategic commitments were impacted by Covid-19	We coded companies on the extent to which such strengthening was evident with reference to three dimensions. The first was managerial mindsets, given that our preliminary analysis had foregrounded how some managers saw in the crisis a signal to give more attention to systemic risks. The second was innovating to meet emergent social or environmental needs during the crisis, including responses identified by Bapuji, de Bakker, and colleagues (2020), although we discounted those measures that were evidently short-lived. The third dimension was collaborating, as some companies showed significant commitment and ability in partnering with diverse actors during the pandemic.	Coded as follows: 1. predominantly restricting sustainability efforts or investments across each of the three dimensions; 2. mostly restricting and perhaps some strengthening in one of the dimensions; 3. significant strengthening in at least one of the dimensions and some strengthening in one other dimension; 4. significant strengthening in at least two dimensions.

Finally, our outcome measure was sustainability strategic response, focused on whether either a strengthening or a restricting response was identified in a company. Companies were coded as strengthening if there was evidence of stronger managerial attention to sustainability issues and investments in sustainability-related innovations and / or collaborative efforts to address social–ecological problems. Conversely, companies were coded as restricting if managers emphasized less focus on sustainability and if investments and collaborative efforts were constrained during the crisis. We coded our interview data and corporate reports for this attribute, again using a 4-point scale. Table 2 provides an overview of our coding and categorization approach for each of the five attributes as well as the outcome. We calibrated the attributes and outcome using the direct method, as per the guidelines set out by Duşa (2019). Our calibration thresholds were exclusion at 1.5, cut-off at 2.5, and full inclusion at 3.5. Table 4 shows the calibrated dataset used to carry out the analysis.

Using the QCA package in R, our analysis aimed to determine the configurational paths that lead to strengthened or restricted sustainability strategic responses. We created truth tables for the outcome and its negation using an inclusion cut-off of 0.8 and a consistency cut-off of 0.5 (Greckhamer et al., 2018; Ragin, 2008). (The truth tables are available on request.) This analysis indicated that four configurations lead to a strengthened sustainability strategic response while six configurations lead to a restricted response. We carried out complex and parsimonious minimizations to determine the most parsimonious solution as well as an intermediate solution (Duşa, 2019).

Using the combination of the intermediate and parsimonious solutions, we were able to compile a configuration table (Fiss, 2011; Ragin, 2008) indicating core and complementary attributes for the configurational pathways that had been arrived at through the minimization process (Table 3). Following convention (Fiss, 2011; Ragin, 2008), solid circles represent the presence of an attribute in the configuration, while circles with a cross in them represent the absence of an attribute. Large circles indicate core attributes, which are those attributes indicated in the parsimonious solution, and small circles indicate complementary attributes, which are identified by comparing the intermediate solution with the parsimonious solution. Empty cells in Table 3 indicate that it does not matter if the attribute is present or not.

**Table 3.** *Configurations Table*.

	**Strengthening**			**Restricting**		
	Governance gap-filling	Building on strengths		Bunkering down	Low-road	Hard hit
**Attributes**	1	2	3	4	5	6
Crisis severity	●	●		⊗	⊗	●
Resource slack	•	●	●	•	⊗	⊗
Sustainability maturity		●	●		⊗	●
Governance stability	⊗		•		●	•
Governance trend			●	⊗		⊗
**Configuration parameters**						
Consistency	0.81	0.81	0.96	0.94	0.90	0.77
Raw Coverage	0.33	0.50	0.22	0.53	0.35	0.21
Unique Coverage	0.05	0.21	0.13	0.37	0.18	0.08
Overall Solution Consistency	0.82			0.96		
Overall Solution Coverage	0.68			0.79		

*Note.* Large characters indicate core conditions. Small characters indicate peripheral conditions. Blanks indicate “does not matter.”

● indicates presence of a condition.

⊗ indicates its absence.

We used the results from the fsQCA to return to our qualitative data to explore and elaborate the underlying dynamics and mechanisms (Misangyi et al., 2017). We analyzed how each case represented the patterns suggested in the associated configurational pathway, recognizing that some cases were included in more than one pathway (because of the empty cells in Table 3). We also compared across cases included in particular pathways to identify the most salient patterns. The point was not to determine exclusive categories of companies but rather to identify the most salient configurational patterns in explaining the outcomes, which sometimes overlapped for specific companies. This resulted in two explanations for “strengthening” responses (one of which combines two of the columns in Table 3), and three explanations for “restricting” responses, as we describe below. Table 4 provides the cases’ association with specific pathways identified in the fsQCA and the corresponding, predominant crisis response dynamic.

**Table 4.** *Overview of Calibrated Data Set and Companies’ Predominant Sustainability Strategic Response Dynamic*.

Company	Crisis severity	Resource slack	Sustainability maturity	Governance stability	Governance trend	Sustainability strategic response	Predominant crisis response
ZA1	3	3	3	66	1	4	Building on strengths
ZA2	2	2	3	66	1	3	Building on strengths
ZA3	1	3	4	66	1	2	Bunkering down
ZA4	4	2	2	66	1	2	Hard hit
ZA5	3	3	3	66	1	2	Bunkering down
ZA6	2	2	1	66	1	1	Low-road business-as-usual
MU1	4	3	2	77	1	2	Hard hit
MU2	2	3	3	77	1	2	Bunkering down
MU3	2	2	3	77	1	3	Building on strengths
MU4	2	3	2	77	1	1	Low-road business-as-usual
MU5	1	3	2	77	1	1	Low-road business-as-usual
MU6	3	3	2	77	1	2	Low-road business-as-usual
MU7	4	2	3	77	1	1	Hard hit
MU8	2	3	2	77	1	2	Low-road business-as-usual
MU9	2	3	2	77	1	1	Low-road business-as-usual
MU10	2	2	2	77	1	3	Gap-filling
NG1	2	4	4	46	1	3	Building on strengths
NG2	2	4	3	46	1	2	Bunkering down
NG3	2	3	2	46	1	4	Gap-filling
NG4	2	4	4	46	1	3	Building on strengths
KE1	2	3	3	59	5	3	Building on strengths
KE2	2	3	3	59	5	3	Building on strengths
KE3	2	2	1	59	5	1	Low-road business-as-usual
KE4	3	1	1	59	5	2	Hard hit
KE5	4	2	2	59	5	2	Hard hit

## Findings

We identified two explanations for “strengthening” responses: “building on strengths” characterized those companies that made use of high sustainability maturity and resource slack to expand their sustainability commitments during the crisis, essentially strengthening their pre-crisis trajectory; and “governance gap-filling” characterized strengthening efforts motivated by a lack of governance stability and effectiveness, with companies seeking to fill governance gaps to ensure their own resilience or to respond to humanitarian needs exacerbated by the crisis. On the flip side, we identified three explanations for “restricting” responses: The “hard hit” explanation applied to companies that were directly and severely impacted by the crisis and did not have significant resource slack to cushion the blow (although they might have had high sustainability maturity); “low-road business-as-usual” dynamics applied to companies that had low sustainability maturity and also limited resource slack, so they had little motivation or ability to expand their commitments (whatever the crisis severity); and, finally, “bunkering down” explains how some companies restricted their efforts because they saw in the crisis an increase in unpredictability exacerbated by already deteriorating governance conditions.

### Strengthening Responses: “Building on Strengths” and “Governance Gap-Filling”

“Building on strengths” is a mechanism through which companies with high sustainability maturity and resource slack continued to expand their sustainability commitments and efforts during the crisis. As illustrated by Columns 2 and 3 in Table 3, the distinctive pattern is the existence of both resource slack and sustainability maturity. As indicated in Column 2, the existence of significant crisis severity did not impede such strengthening and may even catalyze it (as will be discussed), but as shown in Column 3, such direct crisis impacts were not necessary, especially if companies’ governance context is stable and improving. The companies manifesting this dynamic included ZA1, ZA2, MU3, MU10, NG1, NG4, KE1, and KE2.

For example, ZA1 was an alcoholic beverage company with resource slack built up through well-established brands and a leading market position. It also had a mature sustainability strategy and corresponding practices. The sustainability manager had been working there for more than 5 years and had some success in establishing ambitious corporate targets that were directly linked to the Sustainable Development Goals (SDGs), and these targets were connected to senior managers’ key performance indicators and performance bonuses. Executives and board members had been enrolled in taking ownership of particular SDGs, based on a comprehensive socialization process. As noted by the sustainability manager,

One thing that really helped was getting the Board, Exco, to become SDG champions. Many of them didn’t know [initially] what it meant and they were chugging along. We then created cheat-sheets for them, and we put them into the public domain to speak about this. (ZA1 Interview 2)

The company had also established or participated in diverse cross-sector partnerships on sustainability concerns, with a particular emphasis on alcohol abuse, road safety, and gender-based violence. These prior sustainability efforts helped the company respond proactively and expansively during the Covid-19 crisis, providing both the motivation and the capacity to do so. For a start, senior managers’ extant mindsets motivated and enabled them to connect the company’s commitment to the SDGs to the short- and long-term challenges related to Covid-19. Our interviewee noted,

Silver lining of COVID, it gave sustainability a lot of focus. . . with crisis after crisis, Day Zero [the Cape Town drought in 2017-18], femicide, now the pandemic—we know that these crises are becoming more frequent, so the risks are becoming greater [. . . and so our sustainability strategy] becomes more important. (ZA1 Interview 2)

The company was able to make use of existing relationships with the government and other stakeholders to rapidly establish cross-sectoral efforts in response to the crisis, including in particular humanitarian relief in marginalized communities, where the government lockdowns were escalating hunger (Nicolson, 2020). In a similar vein, KE2 was a bank in Kenya that had, prior to the Covid-19 crisis, elevated sustainability as a principal risk with board-level accountability and corresponding key performance indicators for managers. This orientation and related capabilities and relationships were put to good use during the crisis through, for example, collaboration with other companies in the development and provision of psychosocial support to employees.

Our finding on “building on strengths” was not very surprising, as it aligns with prior research which suggested that resource slack and sustainability maturity provide companies with the financial resources and the prior strategic orientation and innovation ability to maintain or expand, rather than restrict, their sustainability efforts (Bansal et al., 2015; Delmas & Pekovic, 2015; Panwar et al., 2015), with links also to the broader crisis management literature (Ocasio, 1995; Williams et al., 2017).

On the contrary, we also came across a different pattern, in which even companies without strong sustainability maturity strengthened their sustainability efforts during the crisis. As is shown in Column 1 in Table 3, the distinctive feature of this configuration is the absence of governance stability and effectiveness at the national level, which led companies to endeavor to fill these gaps during the crisis. This was most notable in MU10 and NG3, where some strengthening occurred despite an absence of sustainability maturity. However, similar motivations were also discernible in some companies with high sustainability maturity, such as NG1 and NG4, in which the “building on strengths” dynamic was complemented by the motivation to fill governance gaps.

There are two reasons for companies seeking to fill governance gaps that were exacerbated by the crisis. The first is to compensate for the state’s inability to provide public goods and services that a company relies upon. For example, NG3 was a fairly young company in Nigeria that had only a nascent sustainability strategy, with very few related commitments, investments, and human resources, but nevertheless, it expanded its sustainability commitments during the crisis. This was in large part because of the recognition that such investments could improve the company’s independence from the state’s inability to provide public goods and services, and this recognition had become more profound during the crisis. To illustrate, just prior to the pandemic, the company had started installing solar energy generation at some of their facilities, instead of diesel generators. This was motivated not only because of environmental reasons but also because of the state’s inability to provide energy to those facilities, as well as the state’s inability to ensure security in some parts of the country, which creates security risks for the generator refueling teams. These governance gaps related to energy and physical security became even more pronounced during the Covid-19 crisis, so the broader business benefits of judicious sustainability-oriented investments became evident also to those company leaders who were not very committed previously. Our interviewee noted that in the crisis, “Suddenly people [i.e., company leaders] woke up to the sustainability topic” (NG3 Interview 2), and the sustainability managers found themselves much more centrally involved in the company’s decision-making processes. As a consequence, broader changes in the strategies and operations of the firm were catalyzed. For instance, the sustainability manager explained, “We started looking at how the whole diesel process, apart from destroying the environment, is also not very sustainable [for the firm]” (NG3 Interview 2).

A different rationale for “governance gap-filling” was to address the humanitarian needs that escalated during the crisis, and which especially states with low governance stability and effectiveness struggled to address. For example, in Nigeria, NG1 responded to the crisis by leveraging existing relationships to address social problems that were amplified by the crisis, including the issue of gender-based violence. As explained by NG1’s sustainability manager: “We have hotlines where we provide people with support for mental wellbeing, giving them access to therapy. From that, we knew that for a lot of women and some men, domestic violence heightened during the lockdown” (NG1 Interview 2). In response, the company developed a national initiative on this theme. The scope and scale of this effort demonstrate how some companies sought to provide public goods and services that would otherwise be expected from the state, even if the underlying social need or the associated public goods and services are not directly linked to the company’s business (although there may be brand and reputation benefits).

### Restricting Responses: “Hard Hit,” “Low-Road Business-as-Usual,” and “Bunkering Down”

“Hard hit” effects are those experienced by companies that were severely and directly affected by the pandemic and associated government lockdown policies, and which had limited resource slack (as indicated by Column 6 in Table 3). These included in particular companies in the travel and tourism sectors, including ZA4, MU1, MU7, and KE5. For example, at MU1 we were told, “there has been a heavy impact [from Covid-19] and we had to cut down in terms of operational excellence [in our sustainability efforts]” (MU1 Interview 2). These restrictions were often perceived as deeply frustrating for our participants, especially when they were curtailing or setting back hard-won achievements in advancing the company’s sustainability strategy before the crisis. For example, our participant at ZA4 explained as follows: “[My sustainability projects] have basically been pushed out because of budget [constraints]. . . there are definitely some restrictions in my world. . . [there’s] nothing about improving the sustainability infrastructure at the moment” (ZA4 Interview 2).

A second, separate set of restricting dynamics is what we refer to as “low-road business-as-usual.” This was visible in companies that were not necessarily severely affected by the crisis, but which were characterized by low sustainability maturity (see Column 5 in Table 3). Relatively little attention was given to sustainability before the crisis, and if anything, the crisis and the associated, immediate priorities further diminished this attention. This effect was evident in ZA6, MU4, MU5, MU8, MU9, and KE3. A telling feature of this dynamic was that participants emphasized cuts to their sustainability budgets, although the company was not very directly or severely affected by the Covid-19 crisis. For example, MU5 was a technology company that did relatively well, financially speaking, during the crisis, yet our participant there noted: “Well honestly, the pandemic has delayed the launch of our [sustainability] goals. . . and has broken the momentum. . . today sustainability is not a priority, with Covid. . . budget is more important” (MU5 Interview 1). Because there were no strong sustainability strategy and corresponding human resources in these companies precrisis, there was little motivation or capacity to advance sustainability during the crisis. For example, at ZA6, the sustainability manager’s position had only been created in mid-2020, so she was faced with the double challenge of developing a sustainability strategy for the company with long-term objectives while most other members’ attention was on short-term priorities related to the Covid-19 crisis. She noted, “working on issues of sustainability as opposed to issues of direct survival is difficult [in the current context]” (ZA6 Interview 1).

The final configurational pattern was more surprising to us. It involved restricting responses even though the direct crisis impact was not severe and regardless of sustainability maturity (see Column 4 in Table 3). The fsQCA suggested that this pathway involved a deteriorating governance context, but on its own this was not very decisive, given that three of our four countries represented this situation. By reanalyzing our qualitative data, we realized that in these companies, company-specific developments combined with concerns about the broader governance context to give rise to an interpretation of the crisis as an uncertain and unpredictable, yet significant threat, which in turn leads to what we call a “bunkering down” response. So, even if the crisis impact was not severe in a direct sense, and even if some sustainability maturity was present, the overarching response was to restrict sustainability efforts. This was visible in ZA3, ZA5, MU2, and NG2.

For example, ZA3 was a retailer with high sustainability maturity and, as an “essential service,” it was not severely or directly affected by the lockdown regulations and its sales were good. Nevertheless, there were strong restricting elements in its approach to sustainability in the crisis. The sustainability manager explained that relative to precrisis conditions, he was “competing much more rigorously for resources [within the firm] than you would necessarily in the past” (ZA3 Interview 2). We argue that an important reason for this was that the Covid-19 pandemic increased already significant concerns about the company’s broader operating environment in South Africa, including both economic and governance dimensions, as expressed by the CEO in prior interviews and in annual reports. Given these concerns around a lack of predictability in the company’s economic and governance context, which were exacerbated by the crisis, there was a general tendency to restrict investments and resource allocation to the firm’s sustainability efforts.

A similar anticipatory dynamic was evident in ZA5, an insurance company with a reputation for progressive sustainability efforts. The direct effect of the Covid-19 crisis was limited in this company during the period of our study. But there was significant uncertainty around what these impacts might be, especially with regard to the company’s possible obligations to pay out for business interruption claims among clients that were directly affected by the lockdown regulations, such as restaurants. Throughout the period of our study, executives’ attention was focused on the legal disputes on this issue unfolding in the courts. Attention to sustainability issues thus languished, and furthermore, the uncertainty around possible impacts of the crisis curtailed the motivation and ability to expand sustainability commitments and investments.

## Discussion

We have argued that it is important to understand the impact of crises on companies’ sustainability strategies, as companies may restrict their sustainability strategies to enhance their own resilience at the organizational level while “undermining planetary survival” (Adams & Abhayawansa, 2021); or alternatively, companies may strengthen their sustainability commitments to build both organizational and societal resilience (DesJardine et al., 2019). We thus contribute to the broader literature on organizational responses to crises (e.g., Chattopadhyay et al., 2001; Dutton, 1986; James et al., 2011; Ocasio, 1995; Osiyevskyy & Dewald, 2018; Sarkar & Osiyevskyy, 2018; Shimizu, 2007; Staw et al., 1981) as well as a more nascent focus on crises’ effects on companies’ sustainability strategies and practices (Bansal et al., 2015; M. L. Barnett et al., 2015; Delmas & Pekovic, 2015; Panwar et al., 2015). We focus specifically on the role of Covid-19 as a particularly severe and widespread crisis with diverse impacts in space and time (e.g., Adams & Abhayawansa, 2021; Bapuji, Patel, et al., 2020; Brammer et al., 2020; Howard-Grenville, 2021).

Our analysis extends understanding of organizational responses to crises in three ways: first, by attending to configurational effects; second, by elaborating how prior investments shape crisis responses; and third, by foregrounding the role of governance contexts. We discuss each of these in turn.

### Understanding the Configurational Effects of Crises

Our first contribution is to highlight and demonstrate the benefits of adopting a configurational approach in explaining the effects of crises on organizational change or rigidity. The prior literature has shown that crises can both enable and constrain organizational change and innovation and that relevant contingent factors include crisis severity, companies’ prior experiences and capabilities, and managers’ mindsets. But because these factors are considered largely independent of each other, extant analyses have tended toward generalized and monolithic arguments. For instance, a crisis that is severe and relatively sudden, as Covid-19 has been for many companies, would thus be assumed by much of the prior literature to lead to threat-rigidity responses (Garretsen et al., 2022; March & Shapira, 1987; Osiyevskyy & Dewald, 2018; Shimizu, 2007). How this overarching effect interacts with, say, prior R&D investment (Sarkar & Osiyevskyy, 2018) has received little explicit attention.

A configurational approach can foreground such interaction effects and thus provide a more diverse and nuanced picture. It allowed us to demonstrate how a specific factor can be more or less influential in supporting either strengthening or restricting responses, depending on its combination with other factors. For example, we show how crisis severity leads to restricting responses if it combines with low resource slack or a deteriorating governance context, but it has less impact if offset by high resource slack and prior strategic investments, and it may even contribute to a strengthening dynamic linked to “governance gap-filling” motivations to address humanitarian needs or enhance organizational resilience. These interaction effects between crisis characteristics and organizational, social, and institutional factors give rise to diverse organizational motivations and abilities, and corresponding responses on the strengthening-restricting spectrum. Specifically, we identify two configurations underpinning a strengthening response and three that lead to a restricting response.

### Elaborating the Role of Prior Investments

We found that companies with resource slack and high sustainability maturity have the motivation and capacity to strengthen their sustainability efforts during the crisis (“building on strengths”). Conversely, companies with low sustainability maturity find little motivation and capacity to do so, and the crisis represents a further setback to their efforts (“low-road business-as-usual”). These findings build on extant arguments on how prior investments create an ability to innovate in response to the crisis (Ocasio, 1995; Osiyevskyy & Dewald, 2018). This likely plays a role in the more specific context of companies’ sustainability responses, but we have little knowledge about the details, nor whether there are additional dynamics at play. The literature on sustainability responses has focused on a reluctance to reverse prior commitments (Bansal et al., 2015; Panwar et al., 2015), but while this might explain the absence of restricting responses, there has been limited explanation of how prior sustainability investments enable a strengthening response.

We thus elaborate this line of reasoning by identifying some of the specific mechanisms, through which prior sustainability investments help strengthen sustainability responses during a crisis. We find that preexisting strengths developed through prior sustainability investments included dedicated, knowledgeable, and well-embedded sustainability managers; a broader array of senior managers with some knowledge and appreciation of the role of sustainability in the firm’s strategy; and a range of established relationships with key stakeholders. These preexisting strengths allowed for an interpretation of the crisis as both a need and an opportunity for further sustainability strengthening as well as the means to do so. On the flip side, we show how preexisting sustainability weaknesses lead to the opposite effect in the “low-road business-as-usual” pathway, where the crisis further distracted managerial attention and commitment away from sustainability objectives. We thus offer more detail on how prior investments provide a company with innovation propensity in a crisis context. Specifically, prior sustainability commitments develop managers with knowledge, mindsets, and relationships that become especially salient in catalyzing a strengthening response.

### The Role of Governance Context in Crisis Responses

Our third contribution is to highlight the role of governance context in organizational crisis responses, including the consideration of both governance quality and the trends over time. In the literature on organizational responses to crises, organizations’ external context has been considered primarily as a source of institutionalized repertoires or templates for action (Ocasio, 1995), but it has hardly been considered as a contingent or moderating factor (for an exception, see Panwar et al., 2015). We find that governance context can play an important role in firms’ responses to crisis, specifically with regard to their sustainability efforts. These effects can contribute to either strengthening or restricting responses, depending on the relative importance of static or dynamic aspects of the governance context, and their combination with other contingent factors.

In the “governance gap-filling” configuration, companies are motivated to strengthen their sustainability efforts because of one of two reasons. One is that the crisis is exacerbating risks to the operations of the firm posed by an inability or unwillingness of the state to provide public goods or services or to enforce commonly binding rules. The other is that the crisis is creating widespread suffering that the state cannot mitigate or ameliorate, and so the company is motivated to become engaged in addressing suffering among employees and their communities in broader humanitarian relief efforts.

These findings on strengthening make specific contributions to the nascent conversation on firms’ sustainability responses to crises. First, the extant literature suggests that crises can in some circumstances strengthen sustainability efforts (Bansal et al., 2015), but there has been limited explanation of what motivates such strengthening. By highlighting the way companies may be motivated to address governance gaps exacerbated by a crisis, involving either organizational resilience or humanitarian motives, we propose two such reasons. Second, our findings help connect the literature on crisis effects to the hitherto separate conversation on how “limited statehood” motivates and shapes business sustainability (Amaeshi et al., 2016; Börzel & Hamann, 2013; Hamann et al., 2020; Matten et al., 2005; Scherer & Palazzo, 2011). This connection can help broaden our perspective because crises’ impacts on organizations include not only the direct impacts that are largely foregrounded in the extant literature but also the indirect impacts created via crisis effects on firms’ broader contexts (Gregg et al., 2022), with specific emphasis on the governance context. Third, our discovery that the crisis’ humanitarian impacts catalyzed significant responses in many companies challenges previous findings that such “peripheral” (Panwar et al., 2015) or “tactical” (Bansal et al., 2015) efforts are restricted during crises. Yet our finding is perhaps intuitive in situations where crises create widespread and visible suffering among employees and others—that is, when the crisis is widely perceived to be humanitarian rather than, say, financial.

We also found that firms’ governance context can lead to the restriction of sustainability efforts in the “bunkering down” pathway. The decisive aspect here was not the static governance context, but its change over time. In contrast to assumptions based on the extant literature, even firms that remain largely unscathed by the crisis and have significant sustainability maturity may restrict their sustainability efforts, if company-specific factors and a deteriorating governance context combine with the crisis to create a greater sense of uncertainty, unpredictability, and threat. This adds an important firm-external dimension to the threat rigidity thesis, which emphasizes both the actual experience of hostility and the cognitive dimensions related to perceiving and anticipating threats (Kreiser et al., 2020; Osiyevskyy & Dewald, 2018; Osiyevskyy et al., 2021; Sarkar & Osiyevskyy, 2018; Staw et al., 1981). We thus contribute to the threat-rigidity thesis by highlighting the role of firms’ external context in this cognitive process, with managers more likely to adopt a threat-anticipation orientation when they are, or perceive to be, in a deteriorating governance context.

### Limitations and Opportunities for Future Research

We included a relatively large sample of company case studies for an inductive, qualitative, and data-rich study (Eisenhardt & Graebner, 2007), and we sought to bring analytical rigor by applying fsQCA (Ragin, 1999, 2012) as recommended also for business sustainability research (Crilly et al., 2012; Halme et al., 2020). We thus adopted a “middle-ground” by trying to create rich data on each case but also sufficient cases for comparative analysis using Boolean logic. This leaves gaps on either side of the depth-breadth spectrum. Future research could explore some of the dynamics in our model in more fine-grained, processual detail in particular cases, possibly including ethnographic methods. For example, this might explore how sustainability managers are able to engage in sensemaking and sensegiving (Hahn et al., 2014; Maitlis & Sonenshein, 2010; Sharma & Good, 2013; Sonenshein, 2016), so as to socialize among their colleagues a view of the crisis as an opportunity for strengthening, rather than a rigidity-inducing threat. This would be useful also because we gave relatively less attention to micro-level cognitive and attitudinal factors in our analysis, although they have been emphasized by some authors in the organizations and crisis literature (e.g., James et al., 2011; Jeong et al., 2022). On the other side of the spectrum, future research could collect data on a larger set of companies and test hypotheses building on our results. Such studies with larger samples could also consider other kinds of crises and / or geographic contexts, as well as contingent, such as companies’ varying ownership structures, so as to elaborate the generalizability of our findings.

Finally, while we collected data over a 1-year period and in two rounds of interviews, future research could take a longer-term perspective to study companies’ responses to crisis over some years. This is pertinent given the possibly varying temporal salience of different corporate responses to crises (Wenzel et al., 2021) as well as the varying degrees to which organizational changes during the crisis will “stick” (Seidl & Whittington, 2020). It would be of particular interest to know whether the strengthening or restricting effects that we encountered in our data will have longer term effects on companies’ contributions to addressing increasingly urgent social–ecological problems.

### Practical Implications

Our findings have practical relevance for managers, investors, and policymakers. For a start, it is useful to recognize when and why some companies restrict their sustainability efforts in response to a crisis. Of course, there are limits to what can be done to support companies that have their business severely disrupted by a crisis and have little resource slack to tide them over (i.e., those affected by the “hard hit” dynamic). But focusing attention on the challenges faced by sustainability managers in such companies is nevertheless important. Managers, investors, and organizations such as the UNGC can engage in diverse efforts to ensure “hard hit” companies do not take their restricting measures so far that valuable prior gains are entirely lost. For example, managers can be supported in participating in multi-stakeholder initiatives to exchange insights and foster motivation.

Conversely, understanding when and how companies may in fact strengthen their sustainability efforts is of practical interest. We show how prior sustainability investments allow companies to “build on strengths” when responding to a crisis, and this creates benefits both for the companies and for their stakeholders—at a time when they need it most. This should provide further incentive for managers, investors, and others to motivate for sustainability investments when times are good. Furthermore, such “building on strengths” companies could play a role in motivating and supporting other companies tempted by restricting responses, for example in collaborative platforms such as the UNGC.

“Governance gap-filling” is also best encouraged and enabled through collaboration platforms, given that the governance gaps affect all companies and the impacts of companies’ gap-filling efforts are more likely successful if many of them contribute to this in a coordinated manner. That said, there is a danger that such “governance gap-filling” can displace the state from its statutory responsibilities (Hamann, 2019), so such efforts should, as far as possible, be designed and implemented in collaboration with the state, rather than as a replacement.

## Conclusion

We clarify when and how firms may respond to crisis with either strengthening or restricting responses in their sustainability strategies and efforts, building on prior findings and extant theories. These dynamics may overlap within specific companies, although some are more likely to predominate than others, depending on company-specific factors and governance conditions. Companies strengthen their sustainability strategies in a crisis when they can “build on strengths” created by prior sustainability investments, with a particularly important role for well-embedded sustainability managers that can both motivate and implement responses in the turmoil of the crisis. Companies also strengthen sustainability efforts when they see a need for “governance gap-filling” to ensure organizational resilience or to respond to humanitarian needs. On the contrary, companies are prone to restrict sustainability efforts when the crisis impact is too severe relative to their slack resources; when their prior sustainability investments and commitments were weak; or when a cognitive threat-rigidity response is triggered by the crisis escalating perceptions of risk linked to a deteriorating governance context. Our findings enhance our understanding of organizational responses to crises by attending to configurational effects, by elaborating on how prior investments shape crisis responses, and by foregrounding the role of governance contexts.
